# The prevalence of IgG antibodies against milk and milk antigens in patients with multiple sclerosis

**DOI:** 10.3389/fimmu.2023.1202006

**Published:** 2023-07-10

**Authors:** Rittika Chunder, Thorsten Heider, Stefanie Kuerten

**Affiliations:** ^1^ Institute of Neuroanatomy, Medical Faculty, University of Bonn, Bonn, Germany; ^2^ Clinic for Neurology, Klinikum St. Marien Amberg, Amberg, Germany

**Keywords:** animal-sourced milk, cross-reactivity, milk proteins, multiple sclerosis, myelin antigens, plant-based milk, personalized diet

## Abstract

**Introduction:**

Multiple sclerosis (MS) is a chronic demyelinating disease of the central nervous system (CNS). The pathophysiology of MS is complex and is said to be influenced by multiple environmental determinants, including diet. We and others have previously demonstrated how consumption of bovine milk can aggravate disease severity in MS patients, which can be explained by molecular mimicry between milk antigens and those expressed within the CNS. In this study we set out to identify alternatives to drinking cow milk which might be less detrimental to MS patients who have a genetic predisposition towards developing antibody titers against bovine milk antigens that cross-react with CNS antigens.

**Methods:**

To this end, we screened 35 patients with MS and 20 healthy controls for their IgG reactivity against an array of animal-sourced milk, plant-based alternatives as well as individual antigens from bovine milk.

**Results:**

We demonstrate that MS patients have a significantly higher IgG response to animal-sourced milk, especially cow milk, in comparison to healthy donors. We also show that the reactivity to cow milk in MS patients can be attributed to reactivity against different bovine milk antigens. Finally, our correlation data indicate the co-existence of antibodies to individual bovine milk antigens and their corresponding cross-reactive CNS antigens.

**Discussion:**

Taken together, we suggest screening of blood from MS patients for antibodies against different types of milk and milk antigens in order to establish a personalized diet regimen.

## 1 Introduction

Multiple sclerosis (MS) is the most prevalent chronic inflammatory demyelinating disease of the central nervous system (CNS) (1). The etiology of MS remains unclear (2). Nevertheless, it is generally accepted that a complex interaction between genetic predisposition and environmental factors results in the disruption of immunological self-tolerance to myelin antigens of the CNS (3). Established environmental risk factors for MS include insufficient sun exposure (4), infection with Epstein-Barr virus (EBV) (5) and dietary intake (6). In particular, several studies have comprehensively elucidated how nutrition can act as a possible co-factor influencing the inflammatory cascade in MS patients either directly, by effecting various cellular elements (7) or indirectly, by interacting with the gut microbiota (8).

For example, the beneficial effect of a diet comprised of fish (9), short chain fatty acids (10), fruits and vegetables (11) on reducing the severity of disease progression in either MS or experimental autoimmune encephalomyelitis (EAE), a commonly used animal model of MS, has been suggested by different reports. Furthermore, a significant epidemiological association between consumption of bovine milk and milk products and the prevalence of MS has been established by other researchers (12–14).

A mechanistic link between the potential exacerbation of autoimmune responses in MS patients and the consumption of milk has been suggested by our group and others (15–17). Stefferl et al. previously reported sequence homologies between myelin oligodendrocyte glycoprotein (MOG) and butyrophilin (BTN), a milk fat globule membrane protein (15). We extended this concept of molecular mimicry to dairy proteins as possible etiological factors in the immunopathogenesis of MS by providing evidence of antibody cross-reactivity between another milk protein, casein, and myelin-associated glycoprotein (MAG) (17).

Despite these findings, among others, that highlight the negative effect of dairy intake on the aggravation of MS symptoms (6, 7, 18), milk and milk products have been an integral part of human diet for centuries. Benefits of milk consumption range from adequate intake of calcium and bone mineralization (19, 20) to nourishment with essential amino acids, vitamins, minerals and fatty acids (21, 22).

With most of these studies (23, 24) focusing on cattle-based milk, here we used enzyme-linked immunosorbent assays (ELISAs) as a method to screen for an immunoglobulin(Ig)G response to different types of animal-sourced and plant-based milk in MS patients and healthy controls. Furthermore, we also correlated the IgG titers between specific bovine milk antigens and their potential cross-reactive myelin antigens in MS patients.

## 2 Materials and methods

### 2.1 Human plasma samples

Plasma samples from 35 patients with MS and 20 healthy donors (as controls) were tested for their IgG reactivity to different milk antigens. The research protocol was approved by the Ethics Committee of the FAU Erlangen-Nürnberg, Germany (file 185_18B). The study used pseudonymized data, and infomed written consent was obtained from all patients. Demographics and disease characteristics of the MS patients are mentioned in **Table 1**. Healthy donors were an age of at least 18 years, had provided written informed consent and did not have any diagnosed diseases, in particular no autoimmune or neurological disorders. The MS (55% female, 45% male) and healthy donor cohorts (65% female, 35% male) had a mean age of 38 (± 11.07) and 39.61 years (± 11.34), respectively.

**Table 1:** Demographics of MS patients.

Sample ID	MS type	EDSS score	Age (Y)	Gender	Disease duration (Y)	MS reatment	OCB status	IgG index
1	3	4.0	38	F	20	Fingolimod	–	–
2	1	3.5	50	F	< 1	–	Pos., Type II, 19 bands	13%
3	2	4.5	53	M	1	–	Pos., Type II, 18 bands	0%
4	1	2.5	26	F	7	Dimethyl fumarate	Pos.	51.4%
5	1	3.5	55	F	1	–	Pos., 6 bands	0%
6	1	2.0	35	M	1	–	Pos., Type II, 19 bands	13%
7	3	7.5	45	F	8	–	Pos., Type II, 18 bands	39%
8	2	5.0	19	F	1	–	Pos., Type II, 15 bands	0%
9	1	1.5	36	M	7	–	–	–
10	2	4.5	38	F	5	–	Pos.	0%
11	2	4.0	27	M	< 1	–	Pos., Type III, 15 bands in.	76%
12	1	3.0	38	F	5	Natalizumab	Pos.,	33.2%
13	1	5.0	29	F	< 1	–	Pos., Typ II, 10 bands	0%
14	1	4.5	37	M	6	Fingolimod	Pos.	11.6%
15	1	4.0	21	M	< 1	–	Pos., Type III, 16 bands in.	15%
16	3	4.0	38	M	4	Natalizumab	Pos., Type II, 21 bands	48%
17	1	3.5	28	F	11	Methotrexate	–	–
18	1	1.0	22	M	2	Natalizumab	Pos., Type II, 4 bands	0%
19	3	3.0	42	F	15	–	Pos.	–
20	2	5.5	49	F	< 1	–	Pos., Type II, 13 bands	64%
21	1	4.0	33	F	11	–	Pos.	2.5 (Norm < 0.7)
22	1	3.5	41	M	2	Glatiramer acetate	Pos., Type II, 7 bands	0%
23	1	2.0	43	M	1	Glatiramer acetate	Pos., Type II, 5 bands	0%
24	1	3.5	32	F	< 1	–	Pos., Type II, 10 bands	26%
25	1	2.5	29	M	10	Alemtuzumab	–	–
26	4	4.5	63	F	31	Interferon-β	–	–
27	1	1.5	50	F	1	Glatiramer acetate	–	–
28	1	2.5	51	F	5	Dimethyl fumarate	–	–
29	1	2.5	23	F	1	Dimethyl fumarate	–	–
30	1	2.0	36	M	8	Interferon-β	–	–
31	1	3.0	44	M	5	Natalizumab	–	–
32	1	3.5	38	F	2	Natalizumab	–	–
33	1	1.5	28	F	3	–	–	–
34	4	5.0	58	M	29	Dimethyl fumarate	–	–
35	1	1.0	36	M	2	Cortisone	–	–

1, relapsing-remitting; 2, primary progressive; 3, secondary progressive with relapses; 4, secondary progressive without relapses; EDSS, expanded disability status scale; F, female; M, male; OCB, oligoclonal bands; Pos., positive; Y, years.

For obtaining the plasma samples, approximately 35 mL of venous peripheral blood was collected in lithium-heparinized tubes and diluted 1:1 in Dulbecco’s phosphate-buffered saline (PBS). Peripheral blood mononuclear cells (PBMCs) and plasma were separated as previously described by our group (25). Briefly, PBMCs were separated from other components of the blood in Leucosep™ tubes (#227290, Greiner Bio-One) by density gradient centrifugation using Ficoll-Paque® PLUS (#17-1440-03, Cytiva). Plasma samples were collected, aliquoted and immediately stored at –80°C until further use. Of note, the PBMCs were not used as a part of this current study.

### 2.2 Antigens and their composition

Antigens used in this study were comprised of recombinant proteins, purified proteins, tissue lysate, and store-bought milk. All antigens were from commercially available sources. **Table 2** gives a summary of the different types of antigens used and their corresponding composition. Store-bought mammalian and plant-based milk were directly aliquoted from the package and frozen until further use. The other purified or recombinant proteins were stored as per their manufacturer’s recommendation.

Table 2Sources of milk and their composition.

**Table:** 

Milk				
	Antigen	Source	Composition	
1	Cow milk	Weihenstephan	Average nutritional values for 100 g – fat 3.5 g; carbohydrates 4.7 g; protein 3.5 g; salt 0.13 g; calcium 120 mg	
2	Goat milk	Andechser Natur	Average nutritional values for 100 g – fat 3.2 g; carbohydrates 4.4 g; protein 3.1 g; salt 0.2 g	
3	Sheep milk	Leeb Vital	Average nutritional values for 100 mL – fat 6.0 g; carbohydrates 4.4 g; protein 4.6 g; salt 0.136 g	
4	A2 milk	Fleckvieh Hof Kraus	Average nutritional values for 100 mL – fat 3.8 g; carbohydrates 4.8 g; protein 3.3 g; salt 0.13 g; calcium 120 mg	
5	Coconut milk	Alpro GmbH	Water; coconut milk (5.3%); rice (3.3%); calcium; stabilizers; sea salt; flavourings; vitamins	
6	Cashew milk		Water; cashew (3.1%); sugar; calcium; sea salt; stabilizers; emulsifier; vitamins	
7	Almond milk		Water; almond (2.3%); sugar; calcium; sea salt; stabilizers; emulsifier; natural flavouring; vitamins	
8	Hazelnut milk		Water; sugar; hazelnuts (2.8%); calcium; sea salt; stabilizers; emulsifier	
9	Oat milk		Water; oat (9.8%); soluble corn fiber; sunflower oil; calcium; sea salt; stabilizers; vitamins

**Table:** 

Purified antigens				
	Antigen	Source	Purification method	Purity
10	α-lactalbumin	L5385 (Sigma-Aldrich)	–	Purity of > 85%
11	β-lactoglobulin	L3908 (Sigma-Aldrich)	Purified by chromatography	Purity of > 90%
12	α-casein	C6780 (Sigma-Aldrich)	Purified by chromatography	Purity of > 70%
13	β-casein	C6905 (Sigma-Aldrich)	–	Purity of > 98%
14	κ-casein	C0406 (Sigma-Aldrich)		Purity of > 70%
15	Whole brain lysate	NB820-59177 (Novus Biologicals)	Total protein prepared from whole tissue homogenates	–
16	Recombinant MOG	8535-MO-050 (R&D Systems)	Expressed in mouse myeloma cell line	Purity of > 95%
17	Recombinant MAG	13186-H08H (Sino Biological)	Expressed in HEK293 cells	
18	Recombinant BTN1A1	BT1-H5222 (Acro Biosystems)		

BTN1A1, butyrophilin; HEK, human embryonic kidney; MAG, myelin-associated glycoprotein; MOG, myelin oligodendrocyte glycoprotein.

### 2.3 Serial dilution and bicinchoninic acid assay

To determine a suitable coating concentration of the different types of store-bought milk, a ten-fold dilution series was carried out. Aliquots of the different types of milk were directly diluted in PBS and a total of 20 dilutions were prepared between 10^-1^ to 10^-20^. Bicinchoninic acid assay (BCA) was done for each dilution using the Pierce^™^ BCA Protein Assay Kit (#23225, ThermoFisher) following the manufacturer’s instructions (microplate protocol). The optical density (OD) values were subsequently measured at 562 nm using a MRXII microplate reader (Dynex Technologies) with Dynex Revelation software (version 4.22). The total protein concentration (in μg/mL) for each of the dilutions/antigen was extrapolated from a standard curve (summarized in **Supplementary Tables S1, S2**).

### 2.4 Enzyme-linked immunosorbent assay

High binding 96-well flat bottom microplates (#655061, Greiner Bio-One) were coated with 100 µL/well antigen (diluted in PBS) and incubated at 4°C overnight. Every antigen was coated in triplicates. The coating concentration of the different antigens is listed in **Table 3**. Wells were blocked with 200 µL/well blocking buffer (10% fetal bovine serum in PBS supplemented with 0.05% Tween-20) at room tempertature (RT) for 3 h. 100 µL of plasma samples diluted 1:100 in PBS-T were subsequently added to the wells and the ELISA plates were incubated overnight at 4°C. Of note, taking the 1:1 pre-dilution of whole blood into account, the final dilution of plasma was 1:200. 100 µL of biotinylated goat anti-human IgG (#A18821, Invitrogen) at a dilution of 1:10,000 in PBS-T was added to every well and the plates were once again incubated at 4°C overnight. Finally, 100 µL of streptavidin-alkaline phosphatase (ALP) (#21324, ThermoFisher) diluted in PBS (1:1,500) was added to the wells and incubated for 2 h at RT. Plates were developed for 60 minutes using the p-nitrophenyl phosphate (pNPP) ELISA substrate (#3652-P10, Mabtech) and the absorbance (OD) was recorded at 405 nm using a microplate reader (Infinite® M200, Tecan). Plates were washed at least three times with either PBS or PBS-T between every incubation step. Negative controls did not contain any plasma sample and were included on each plate. All plates had the same lot number. However, to ensure low interplate variability, three plates were coated with three different antigens (cow milk, goat milk and β-casein) and tested against eight randomly selected MS and four healthy donor plasma samples. Plasma samples of two healthy donors with a high titer against EBV were additionally tested against EBV antigen on each of the three plates. The interplate coefficient of variation for the two healthy donors tested against EBV was 0.033% and 0.000472%, respectively. In addition, this experiment confirmed the interassay stability of the milk antigen-specific response for a given set of representative healthy donors and MS patient samples. The interassay coefficient of variation was in the range of 0.101 – 0.00366% for cow milk, 0.0181 – 0.000019% for goat milk and 2.56 – 0.00193% for β-casein.

**Table 3:** Antigens used and their dilutions.

	Antigen	Species/type	Total protein coating concentration
1	Cow milk	Animal-sourced	30.88 – 35.71 μg/mL
2	Goat milk		
3	Sheep milk		
4	A2 milk		
5	Coconut milk	Plant-based	
6	Cashew milk		
7	Almond milk		
8	Hazelnut milk		
9	Oat milk		
10	α-lactalbumin	Bovine	10 μg/mL
11	β-lactoglobulin		
12			
13	α-casein		
14	β-casein		
15	κ-casein		
16	Whole brain lysate	Human	
17	Recombinant MOG		1 μg/mL
18	Recombinant MAG		
19	Recombinant BTN1A1		

BTN1A1, butyrophilin; MAG, myelin-associated glycoprotein; MOG, myelin oligodendrocyte glycoprotein.

### 2.5 Statistics

GraphPad Prism 8.0 (GraphPad Software, Inc.) was used for statistical analysis. A Shapiro–Wilk normality test was used to verify Gaussian distribution of the data sets. Since none of the data sets were normally distributed, a Mann-Whitney test with a significance level of 5% was used. Spearman’s correlation coefficient was used to determine the association between two or more variables.

## 3 Results

### 3.1 MS patients exhibit a significantly higher IgG response to cow milk and goat milk compared to healthy donors

In the first set of experiments, we quantified and compared IgG reactivity to different sources of mammalian and plant-based milk in MS patients and healthy donors. Antigen coating concentrations between 31 – 36 μg/mL (corresponding to 10^-15^ in the row of serial dilution) were used against a standard plasma dilution of 1:200 for all patients and controls (**Table 3**).

**Figure 1:**
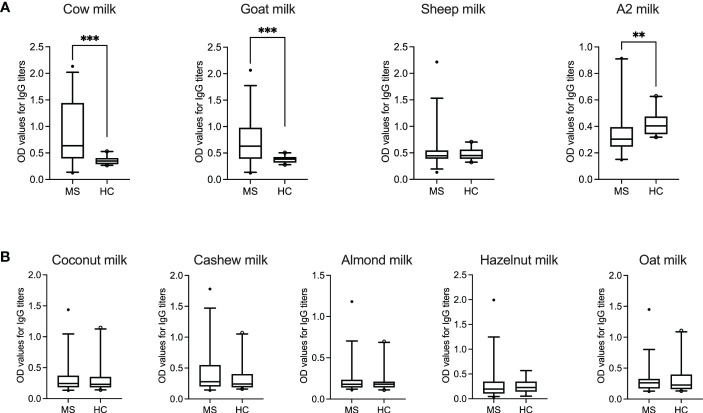
IgG response to animal-sourced and plant-based milk in MS patients and healthy controls. OD values corresponding to IgG titers against **(A)** animal-sourced milk (i.e., cow, goat, sheep and A2 milk) and **(B)** plant-based alternatives (i.e., coconut, cashew, almond, hazelnut and oat milk) are shown in MS patients (*N* = 35, except for hazelnut milk where *N* = 34) and healthy donors (*N* = 20, except for hazelnut milk where *N* = 13). ODs represent the mean of triplicates for every plasma sample. Median with 5-95 percentile range is displayed in the graph. **P < 0.005; ***P < 0.001. HC, healthy controls; MS, multiple sclerosis; OD, optical density.

As shown in **Figure 1A**, IgG titers to cow milk (*P* = 0.0002; Mann–Whitney test) and goat milk (*P* = 0.0004; Mann–Whitney test) in patients with MS were significantly higher than in healthy donors. Furthermore, while the IgG response to mammalian (in particular, cow and goat) milk was very heterogenous between the individual MS patients, there was less variation or dispersion in the individual OD values of healthy donors.

Interestingly, healthy controls displayed a significantly higher IgG response to A2 milk (*P* = 0.0032; Mann–Whitney test) compared to the MS cohort. The “A2” in A2 milk refers to one of the two variants of bovine β-casein, which differs from A1 β-casein (present in regular cow milk) by one single amino acid (26). A bioactive peptide, β-casomorphin-7 (BCM-7), which has cytomodulatory properties, can be released by digestion of A1 β-casein but not A2 β-casein (27).

No statistical significance was achieved between the two groups for IgG reactivity against sheep milk. Similarly, the IgG response to the different plant-based milk alternatives was comparable in both patient and control groups (**Figure 1B**). **Table 4** summarize the range, standard deviation and mean OD values for the different types of milk in both MS and healthy control cohorts. **Supplementary Figure S1** categorizes the mean (± SD) OD values (corresponding to the IgG titers to the different categories of milk) based on gender.

**Table 4:** Summary of the optical density (OD) values of the different milk types for MS patients and healthy controls.

Antigen	Cohort	Range (OD)	Mean (OD)	± SD (OD)
Cow milk	MS	2.132 – 0.122	0.843	0.575
	HC	0.529 – 0.266	0.356	0.069
Goat milk	MS	2.06 – 0.119	0.769	0.479
	HC	0.507 – 0.278	0.375	0.062
Sheep milk	MS	2.214 – 0.133	0.560	0.379
	HC	0.707 – 0.322	0.472	0.105
A2 milk	MS	0.914 – 0.146	0.353	0.197
	HC	0.628 – 0.318	0.420	0.092
Coconut milk	MS	1.436 – 0.116	0.333	0.263
	HC	1.149 – 0.140	0.319	0.243
Cashew milk	MS	1.781 – 0.132	0.424	0.368
	HC	1.071 – 0.160	0.328	0.222
Almond milk	MS	1.180 – 0.108	0.233	0.192
	HC	0.698 – 0.109	0.224	0.143
Hazelnut milk	MS	1.993 – 0.031	0.324	0.376
	HC	0.568 – 0.053	0.256	0.142
Oat milk	MS	1.449 – 0.117	0.298	0.318
	HC	1.108 – 0.128	0.230	0.244

HC, healthy controls; MS, multiple sclerosis; OD, optical density; SD, standard deviation.

### 3.2 IgG response to cow milk is a result of reactivity to multiple proteins

**Figure 2:**
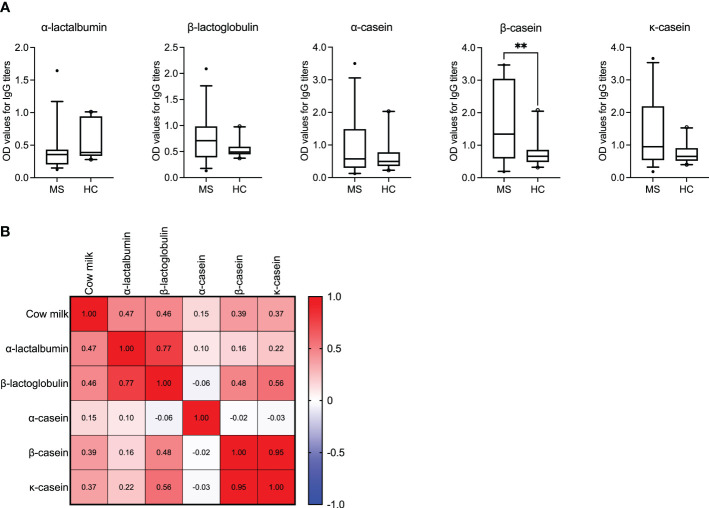
IgG reactivity to bovine milk antigens in MS patients and healthy controls. **(A)** IgG titers against the individual bovine milk antigens are displayed for the MS (*N* = 35) vs. healthy control (*N* = 20) cohorts. Median with 5-95 percentile range is shown. **P < 0.005. **(B)** The correlation matrix displaying the correlation co-efficient (*r*) indicates the co-occurance of antibodies against cow milk and the individual milk antigens in MS patients. OD, optical density.

Given that the statistical significance of IgG reactivity to cow milk was the highest when comparing MS patients and healthy donors, we went on to investigate the protein(s) that could be responsible for the increased antibody titers in the patient cohort. Accordingly, we looked into the antibody reactivity against individual cow milk proteins in the two groups. As shown in **Figure 2A**, the protein antigens chosen were: α-casein, β-casein and κ-casein – which are the three major subtypes of casein found in cow milk and together account for 80% of the total milk protein; α-lactalbumin and β-lactoglobulin as the two other prevelant cow milk proteins are present in the remaining 20% of the protein content (28).

We observed significantly higher IgG titers to β-casein (*P* = 0.0049) in the MS cohort compared to the healthy donors. Additionally, for the antigen β-casein, it appeared that the MS patients could be segregated into two groups based on their mean OD values (± SD) >/< 2.0. Accordingly, we investigated if these patients with OD values >/< 2.0 also had a higher or lower expanded disability status scale (EDSS) score and longer or shorter disease duration, respectively. Results are shown in **Supplementary Figure S2** and imply that higher OD values are associated with higher EDSS scores (*P* = 0.0045). Interestingly, despite the overall low IgG titers to α-lactalbumin in MS patients, the correlation between the presence of antibodies to α-lactalbumin and cow milk was the highest (*r* = 0.47, *P* = 0.004; Spearman’s correlation). A correlation range between *r* = 0.15 and *r* = 0.46 was achieved for the other individual antigens, as shown in **Figure 2B**. The *P* values corresponding to the correlation coefficients are shown in **Supplementary Table S3**.

### 3.3 IgG response to cow milk proteins is a result of possible cross-reactivity to multiple CNS antigens

We have previously shown that reactivity to cow milk casein correlated with a B cell response to a mixture of CNS antigens. We have also demonstrated that the IgG response to casein in these patients could be attributed to cross-reactivity with the myelin antigen MAG (17). Another study reported sequence homology between the CNS myelin-specific antigen MOG and the milk fat globule membrane protein BTN (15, 16).

**Figure 3:**
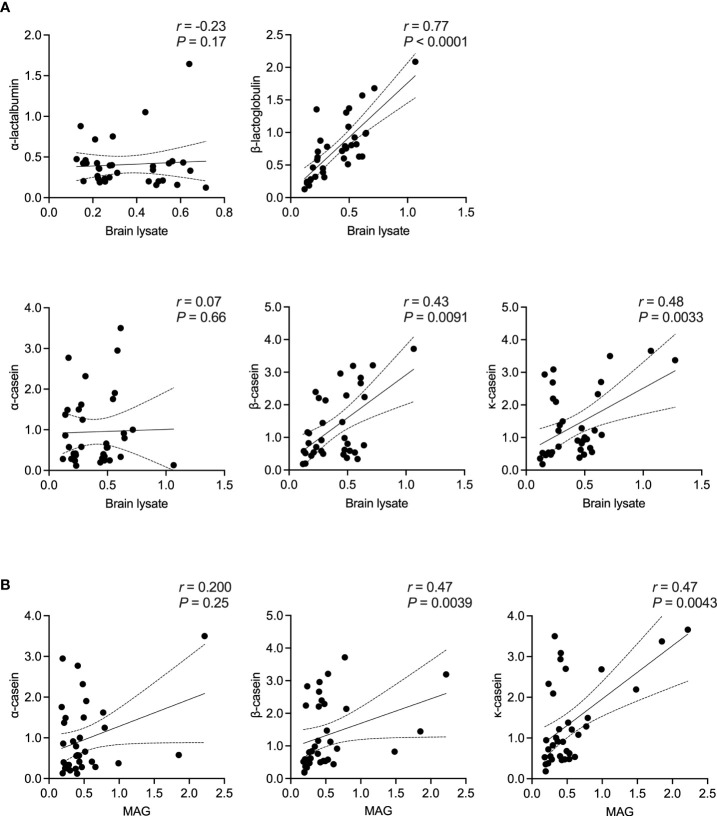
Correlation between bovine milk antigens and CNS antigens in MS patients. Spearman’s correlation between the presence of IgG to **(A)** the different bovine milk antigens vs. brain tissue lysate and **(B)** the individual casein antigens vs. MAG. Correlation coefficient (*r*) and the corresponding significance (*P*) are displayed in the graph. MAG, myelin-associated glycoprotein; OD, optical density.

**Figure 4:**
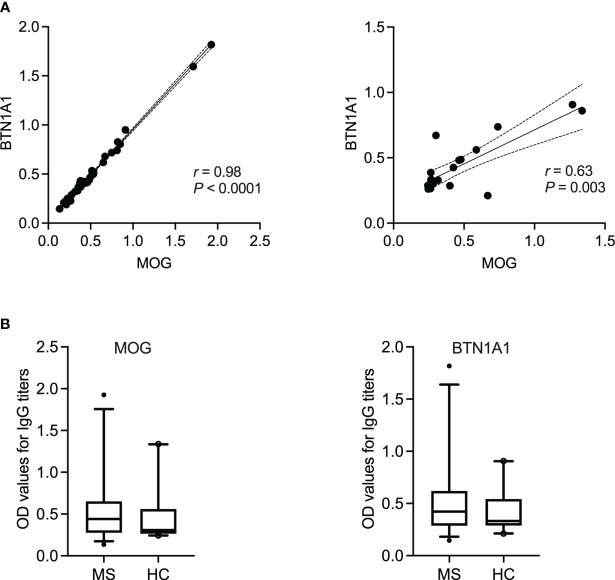
IgG reactivity to BTN1A1 and MOG in MS patients and healthy controls. **(A)** Spearman’s correlation between the occurance of IgG to milk fat globule membrane protein BTN1A1 and MOG in MS patients (*N* = 35) or healthy controls (*N* = 20). Correlation coefficient (*r*) and the corresponding significance (*P*) are displayed in the graph. **(B)** IgG titers to BTN1A1 and MOG are shown for MS (*N* = 35) vs. healthy controls (*N* = 20). BTN1A1, butyrophilin; MOG, myelin oligodendrocyte glycoprotein; OD, optical density.

Here, we first examined the correlation between an antibody response to the different milk proteins and whole brain tissue lysate as a source of CNS antigens. Then, using MAG and MOG as two already identified cross-reactive myelin antigens, a correlation between the individual casein subtypes or BTN1A1 (previously known as BTN), respectively, was established. Of note, no other myelin antigens were included in our study given the lack of evidence for their corresponding cross-reactive milk antigens. As shown in **Figure 3**, the highest correlation in MS patients was observed between an IgG response to β-lactoglobulin and brain lysate (*r* = 0.77, *P* < 0.0001; Spearman’s correlation). For the individual casein subtypes, the correlation was strongest between κ-casein and brain tissue lysate (*r* = 0.48, *P* = 0.0033) and lowest between α-casein and brain lysate (*r* = 0.07, *P* = 0.66). A similar trend was observed when the individual casein subtypes were correlated against MAG, with the with the highest correlation observed between b-casein and MAG (r = 0.47) and κ-casein and MAG (r = 0.47), and the lowest between α-casein and MAG (*r* = 0.20). A very high correlation was observed between BTN1A1 and MOG with correlation co-efficients of *r* = 0.98 and *r* = 0.64 in the MS and healthy control cohorts, respectively. However, both cohorts harbored comparable levels of IgG to MOG and BTN1A1 (**Figure 4**).

## 4 Discussion

Milk proteins, specifically bovine milk antigens, have been shown to share peptide homology with different neuronal and non-neuronal antigens (15–17, 29–32). Therefore, loss of oral tolerance to these ingested milk antigens (33, 34) can lead to aggravation of ongoing autoimmunity as a result of molecular mimicry, as in the case of MS. Indeed, the immune repertoires are greatly heterogenous between individual patients (35) and the possible consequences of this tolerance breakdown only apply to those who already harbor milk-antigen reactive T cells and B cells. Accordingly, the purpose of this study was to analyze the overall prevalence of antibodies against different sources of milk and milk antigens in a cohort of MS patients and correlate these findings to the presence of antibodies against CNS tissue lysate and specific recombinant antigens in the same patients.

To this end, as the first approach, we screened for IgG reactivity to different sources of milk in a cohort of 35 MS patients and 20 healthy donors. Our data indicate that IgG reactivity in MS patients in comparison to healthy donors was highest against cow milk followed by goat milk, whereas the antibody titers to sheep milk were comparable between the two groups. Loss of oral tolerance to specific milk antigens initiated by disturbance in antigen uptake and presentation in genetically susceptible individuals (34) can be an explanation why MS patients harbor higher IgG titers to animal milk than healthy donors. A potential factor could be the disruption of the intestinal epithelial barrier resulting in increased permeability of potentially pathogenic antigens into the body (36). Leaky gut syndrome has been reported to play a critical role in several autoimmune diseases like MS resulting in dysbiosis and alterations in the composition of the gut microbiome (37).

Interestingly, however, our healthy control cohort displayed a higher IgG response to A2 milk (*P* = 0.008) compared to the MS patients. As mentioned earlier, A2 refers to one of the two variants of bovine milk β-casein (26) with reports suggesting that consumption of milk containing only A2 β-casein is associated with fewer gastrointestinal symptoms than conventional milk in those with milk allergies (38, 39). Furthermore, elevated levels of β-casein antibodies in patients with type 1 diabetes (40) have been attributed to the A1 variant of β-casein as the antigenic trigger (41). We observed a similar trend in our cohort where, on the one hand, MS patients harbored significantly higher IgG titers to β-casein (**Figure 2**) in comparison to the healthy controls. Of note, the β-casein antigen used in this study does not specifically contain either only the A1 or A2 variant of β-casein (**Table 2**). On the other hand, the overall antibody reactivity to A2 (cow) milk was significantly lower in the MS cohort. Taken together (**Figures 1**, **2**), therefore, one may speculate that in MS patients the overall reactivity to bovine milk that was observed is, at least partially, due to an elevated IgG response to the A1 β-casein. This would also explain the low OD values to the A2 milk which does not contain the allergen and antigen A1 β-casein. Future studies focusing on adsorption assays using A1 vs. A2 β-casein would validate this observation.

Another finding of this study was that both MS patients and healthy donors showed similar patterns and levels of IgG reactivity to the different types plant-based milk (**Figure 1B**), with reactivity to almond milk being the lowest and to cashew and coconut milk, on average, the highest. Indeed, the main sources of plant proteins that are consumed as an alternative to animal proteins, like nuts, wheat and soy, are considered to be important allergenic triggers (42). On the one hand, healthy subjects have been reported to possess antibodies to coconut, almond and soy-based milk substitutes (43). On the other hand, an increased antibody (IgG) titer to certain plant antigens, like those in soybean and corn, has also been reported in a subset of patients with autoimmune neuroinflammation (44). Furthermore, this reactivity has been attributed to the expression of aquaporins (AQPs) in plants that are known to facilitate the transport of water and sustain plant life (44). Plant AQPs have been shown to share homology with human aquaporins (45, 46), in particular AQP4, which is a known autoantigen in the neuroimmune disorder neuromyelitis optica (NMO) (47).

Vaishnav et al. reported that serum from NMO patients cross-reacted with a sequence found in plant aquaporins which shared homology with the human AQP4 epitope (48, 49). They also demonstrated that this cross-reactivity was higher in NMO patients compared to non-NMO controls (48). Whether the plant-based milk alternatives that were used in this study (i.e., coconut, oat, cashew, almond and hazelnut) also express the human AQP4-cross-reactive pathogenic epitope mentioned by Vaishnav et al. remains to be clarified (48). Furthermore, one can speculate that because MS is not an autoimmune astrocytopathy (50), our patient cohort did not have any significantly higher levels of antibodies to the plant-based milk types in comparison to the healthy donors. Finally, one important point to consider is the low concentration of potentially pathogenic antigen(s) present in the plant-based alternatives. The percentage of plant protein and therefore the relevant antigen(s) present in the different sources of commercially available plant-based milk are < 10% which might not be sufficient to provoke a cross-reactive antibody response in patients with an underlying autoimmunity.

When segregating our data according to gender, we observed no significant differences in IgG titers to the different sources of milk (animal- or plant-based) between male and female MS patients. A similar result was observed also for the healthy donors, except for the reactivity to sheep milk where female healthy controls had a significantly higher titer (*P* = 0.009) in comparison to their male counterpart. However, this finding can also be explained by the relatively low *N* numbers in both the male and female cohorts.

In this study, we investigated the IgG titers against the three most abundantly present bovine milk antigens (51) and observed significantly elevated IgG titers to β-casein (*P* = 0.0049) in the MS cohort. When the MS patients with IgG to β-casein were further categorized into those with an OD value > 2.0 vs. < 2.0, a significance between the two groups in terms of their corresponding EDSS score was observed (**Supplementary Figure S2**), indicating that patients with high titers to β-casein also suffered from a greater disability. Nevertheless, in this study, the dairy intake of the recruited MS patients or the corresponding healthy controls has not been documented. Careful monitoring of milk intake and its effect on the IgG titers to the individual milk antigens needs to be performed in a longitudinal manner to confirm this preliminary data.

We also correlated the overall IgG response against cow milk and the individual cow milk antigens in the MS cohort. The correlation matrix showed the highest correlation between the presence of antibodies to α-lactalbumin and cow milk (*r* = 0.47) followed by antibodies to β-lactoglobulin and cow milk (*r* = 0.46). Why only a moderate linear relationship was observed between a response to casein antigens and cow milk in this study can perhaps be explained by the nature of the different sources of antigens used. For example, the individual bovine caseins (i.e., α-casein, β-casein, κ-casein) that were used in this study were purified antigens. While the source of cow milk was store-bought and therefore the coating antigen did not only comprise purified bovine milk proteins but rather a heterogenous mixture of other unrelated proteins. Therefore, our correlation data needs to be interpreted with caution, also acknowledging that “*correlation does not imply causation*”.

Our data also highlighted that the presence of antibodies to human brain lysate in MS patients strongly correlated with the IgG titer to bovine β-lactoglobulin (*r* = 0.77). On the one hand, there are reports suggesting that β-lactoglobulin shares sequence and structure homology with a number of different antigens expressed in human brain tissues, like glutamate decarboxylase (GAD)-65 (52, 53) and β2-microglobulin (54, 55). Furthermore, both GAD-65 and β2-microglobulin are also involved in different brain diseases like Stiff-Person’s syndrome (56) and glioblastoma (57). On the other hand, β-lactoglobulin is restricted to bovine milk and this protein is not expressed in humans (58), making it a highly allergenic and pathogenic antigen (59), with particular relevance to different human diseases. To what extent the high correlation we detected in our MS cohort can be attributed to antibody cross-reactivity between bovine β-lactoglobulin and GAD-65 and β2-microglobulin remains unknown. Future studies should focus on validating the nature of this antibody cross-reactivity as well as exploring the possibility that there may be other bovine β-lactoglobulin cross-reacting antigens expressed in the brain tissue.

We were able to confirm the simultaneous presence of IgG to bovine casein and MAG in MS patients, where MAG is the cross-reactive antigen for bovine casein (17). Indeed the correlation was moderate but this could be, once again, due to the nature of the antigens used. While the casein antigens were purified by chromatography from bovine casein with a purity between ≅ 70% (for α- and κ-casein) and ≅ 98% (for β-casein), recombinant MAG was used (**Table 2**). Of note, the significant correlation between the occurance of IgG to bovine β-lactoglobulin, β- and κ-casein vs. human brain tissue lysate in the MS cohort was not observed in the healthy control group (**Supplementary Figure S3**). Similarly, there was also no correlation between the presence of antibodies to MAG and the individual casein antigens (especially, β- and κ-casein) in the healthy cohort (**Supplementary Figure S4**).

Finally, the IgG titers to neither BTN1A1 nor MOG were significantly higher in the MS patient cohort in comparison to the healthy controls, which is in line with the literature (16, 60). Indeed, the fact that we did not detect significantly higher titers of anti-MOG antibodies in the MS cohort could be a technical issue since whether these antibodies recognize linear or conformational epitopes of MOG remains under debate. Nevertheless, we observed a very high correlation between BTN1A1 and its cross-reactive antigen MOG (**Figure 4**), in which case both were recombinantly expressed proteins, with *r* = 0.98 and *r* = 0.64 in the MS and healthy cohorts, respectively. This was not surprising given that studies have previously extensively demonstrated that MHC class II-restricted CD4^+^ T cell responses to BTN and MOG are mutually cross-reactive and that BTN can be processed and presented *in vivo* to simulate a cross-reactive T cell response to MOG in a rat model of MS (15). Furthermore, it has also been reported that BTN and MOG cross-react in a subset of MS patients (16). Therefore, the high correlation between IgG to MOG and BTN1A1 in our patient cohort was a successful extension of the data already available since the early 2000s.

While the encephalitogenic properties of BTN have already been discussed (16), cross-reactivity between MAG and casein resulting in CNS demyelination in a mouse model has previously been demonstrated by our group (17). Future studies should focus on further elucidating pathomechanistic processes of potential molecular mimicry, e.g., by passively transfering antibodies against different milk antigens into mice in the context of a compromised blood-brain barrier to determine whether the transfer will exacerbate CNS inflammation and demyelination. This could be done in combination with adsorption experiments to validate the specificity of the antibodies. In addition, further efforts could be made to identify additional self-antigenic targets of milk-specific antibodies.

## 5 Conclusion

Taken together, we hypothesize that consumption of animal-based milk antigens that share sequence or structural homology with human tissue-specific proteins can result in mimicry-induced misfires of the immune system in susceptible individuals (61–64). However, one should not assume that consumption of any plant-based milk alternatives by these patients is automatically a better choice. Therefore, on the one hand our result calls for a thorough testing for the determination of antibody titers against an array of different mammalian and plant-based milks/milk substitutes on an individual basis. On the other hand, removal of such antigens, for example, a dairy-free diet in individuals with a genetic predisposition can be used in the development of personalized medicine.

One of the caveats of this observational study is that the effect of different immunomodulatory therapies and the amount of daily dairy and dairy product consumption on the IgG response to milk and CNS antigens in the MS cohort have not been taken into account. Furthermore, the antibody response has been measured only for a single time point. Therefore, future studies should validate the findings presented in this manuscript using a larger and stratified cohort of MS patients whose IgG titers against the different dietary antigens can be measured in a longitudinal manner, also including paired cerebrospinal fluid (CSF) and serum samples. This will provide a deeper insight into the possible sequestration of milk antigen-specific antibodies into the CSF/CNS compartment. Additionally, the impact of immunomodulatory or B cell depleting therapies on the IgG response to milk antigens needs to be studied in a systemic manner. In the current study, the MS cohort was neither under any special diet nor did the patients suffer from any known milk allergies. Questionnaires provided to the MS patients to document the amount of their daily consumption of animal-sourced milk or vegan milk alternatives will provide crucial information on how diet may have an impact on the immune response to different milk antigens. This will be a necessary step for creating a customized diet plan tailored according to every patient’s need.

Finally, we have previously demonstrated that antibodies to bovine milk casein cross-react with MAG (17). While the current study confirms the correlation between anti-casein and anti-MAG IgG in the MS cohort, we do not have any further knowledge regarding the precise biochemical structure of the cross-reacting epitopes. Whether antibodies developed against cow milk casein(s) target the glycans of MAG, or bind to the protein itself remains to be clarified. For example, in the case of the rare peripheral polyneuropathy, anti-MAG IgM gammopathy, antibodies are specifically directed against the carbohydrate HNK-1 independent of the protein backbone (65). If this would be the case for the anti-casein antibodies where they recognize the glycans rather than the protein, enzymatic deglycosylation of caseins (or other milk-related cross-reactive antigens) before consumption may be an important point to consider.

## Data availability statement

The original contributions presented in the study are included in the article/**Supplementary Material**. Further inquiries can be directed to the corresponding author.

## Ethics statement

The studies involving human participants were reviewed and approved by the Ethics Committee of the FAU Erlangen-Nürnberg, Germany (file 185_18B). The patients/participants provided their written informed consent to participate in this study.

## Author contributions

RC and SK designed research and wrote the manuscript. RC performed the experiments. TH recruited the patients and was responsible for their clinical monitoring. All authors contributed to the article and approved the submitted version.
