# Supplementary material for: The prevalence of IgG antibodies against milk and milk antigens in patients with multiple sclerosis
Source: Front Immunol. 2023 Jul 10;14:1202006. doi: 10.3389/fimmu.2023.1202006 (PMC10364054; doi:10.3389/fimmu.2023.1202006)
Supplement: Supplementary file 1 [file DataSheet_1.docx]

Supplementary Material

The prevalence of IgG antibodies against milk and milk antigens in patients with multiple sclerosis

**Table S1: BCA standard curve.**

| **Standard** | **Duplicate 1 (OD)** | **Duplicate 2 (OD)** | **Average (OD)** | **Concentration (μg/mL)** | **Blank subtracted** |
| --- | --- | --- | --- | --- | --- |
| 2000 | 1.963 | 1.947 | 1.955 | 2000 | 1.8775 |
| 1500 | 1.815 | 1.893 | 1.854 | 1500 | 1.7765 |
| 1000 | 1.345 | 1.322 | 1.3335 | 1000 | 1.256 |
| 750 | 1.063 | 1.0 | 1.0315 | 750 | 0.954 |
| 500 | 0.812 | 0.8 | 0.806 | 500 | 0.7285 |
| 250 | 0.477 | 0.48 | 0.4785 | 250 | 0.401 |
| 125 | 0.296 | 0.304 | 0.3 | 125 | 0.2225 |
| 25 | 0.139 | 0.135 | 0.137 | 25 | 0.0595 |
| Blank | 0.078 | 0.077 | 0.0775 | 0 | 0 |

BCA, bicinchoninic acid assay; OD, optical density.

**Table S2: Protein concentrations of individual antigens estimated from the standard curve.**

| **Antigen** | **OD values corresponding to** | | | **Concentration of antigen corresponding to 10^-15^ (μg/mL)** |
| --- | --- | --- | --- | --- |
|  | **Dil. 1**  **(10^-5^)** | **Dil. 2**  **(10^-10^)** | **Dil. 3**  **(10^-15^)** |  |
| Cow milk | 2.3945 | 0.2375 | 0.076 | 31.93 |
| Goat milk | 2.2935 | 0.2125 | 0.0755 | 31.72 |
| Sheep milk | 2.469 | 0.399 | 0.085 | 35.71 |
| A2 milk | 2.2825 | 0.279 | 0.079 | 33.19 |
| Coconut milk | 1.8915 | 0.199 | 0.0825 | 34.66 |
| Cashew milk | 1.0715 | 0.121 | 0.0815 | 34.24 |
| Almond milk | 1.319 | 0.142 | 0.081 | 34.03 |
| Hazelnut milk | 0.9735 | 0.1475 | 0.0735 | 30.88 |
| Oat milk | 2.1265 | 0.2795 | 0.0845 | 35.5 |

Dil., dilution; OD, optical density.

**
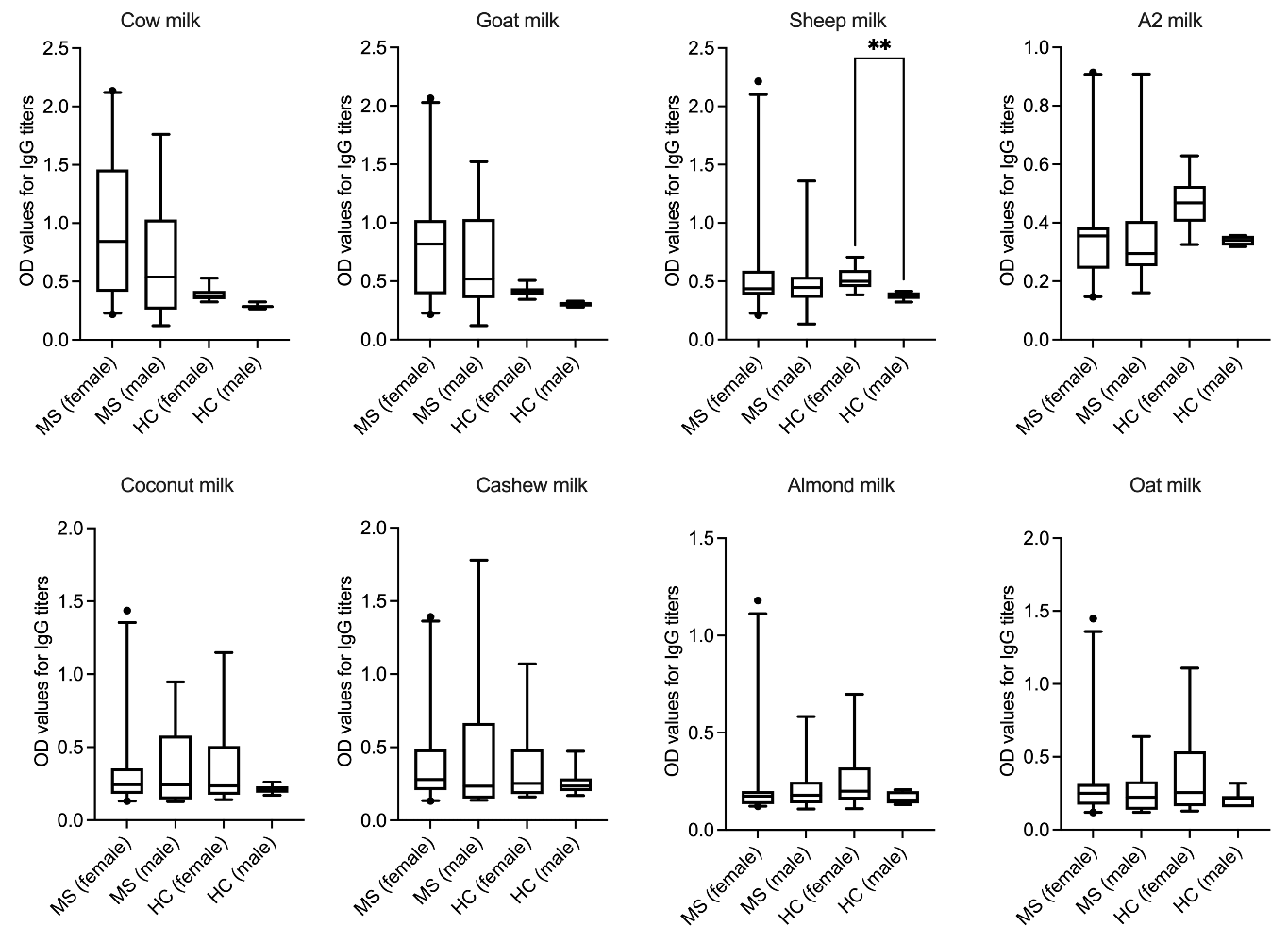
**

**Figure S1 Categorization of OD values for the different types of milk based on gender.** OD values corresponding to IgG titers against **(A)** animal-sourced milk (i.e., cow, goat, sheep and A2 milk) and **(B)** plant-based alternatives (i.e., coconut, cashew, almond, hazelnut and oat milk) are shown in MS patients (*N* = 35, except for hazelnut milk where *N* = 34) and healthy donors (*N* = 20, except for hazelnut milk where *N* = 13) segregated by gender. OD represents the mean of triplicates for every plasma sample. Median with 5-95 percentile range is displayed in the graph. ***P* < 0.005. HC, healthy controls; MS, multiple sclerosis; OD, optical density.


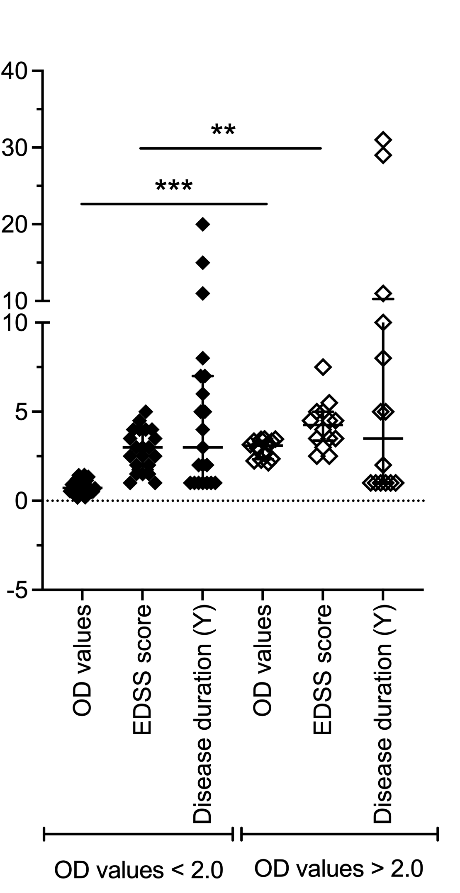


**Figure S2 Categorization of EDSS and disease duration in MS patients based on OD values > < 2.0 for β-casein.** The EDSS score and the disease duration (in years) corresponding to OD values >/< 2.0 to β-casein are shown. Median values with interquartile range are displayed in the graph. ***P* < 0.005; ****P* < 0.001. EDSS, expanded disability status scale; OD, optical density; Y, years.

**Table S3: *P* values corresponding to the Spearman’s correlation matrix in Figure 2B.**

| ***P values*** | Cow milk | α-lactalbumin | β-lactoglobulin | α-casein | β-casein | κ-casein |
| --- | --- | --- | --- | --- | --- | --- |
| Cow milk |  | 0.0043 | 0.0049 | 0.391 | 0.0195 | 0.0301 |
| α-lactalbumin | 0.0043 |  | < 0.00001 | 0.582 | 0.351 | 0.204 |
| β-lactoglobulin | 0.0049 | < 0.00001 |  | 0.731 | 0.004 | 0.00016 |
| α-casein | 0.391 | 0.582 | 0.731 |  | 0.917 | 0.872 |
| β-casein | 0.0195 | 0.351 | 0.004 | 0.917 |  | < 0.00001 |
| κ-casein | 0.0301 | 0.204 | 0.00016 | 0.872 | < 0.00001 |  |

**
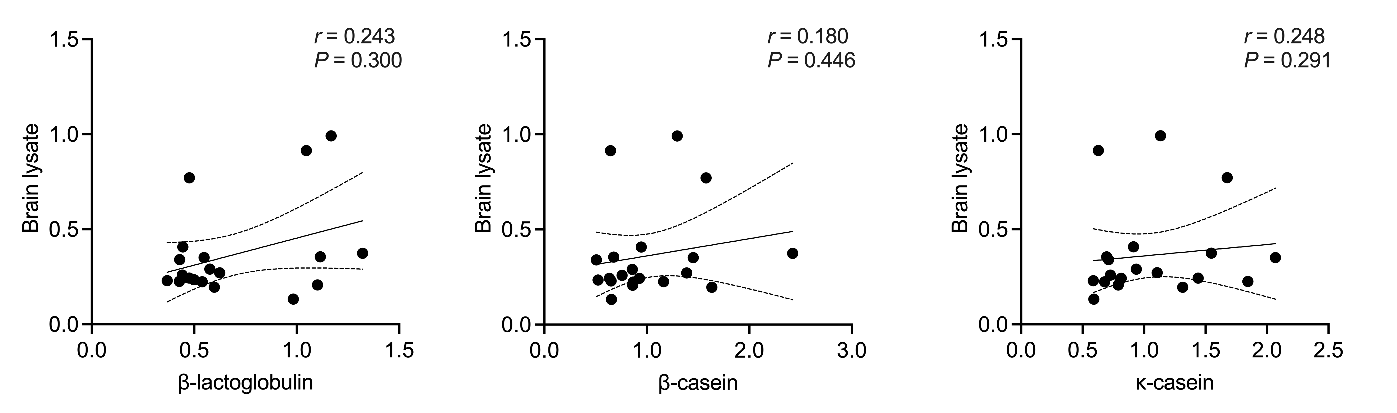
**

**Figure S3 Correlation between β-lactoglobulin. β- and κ-casein *vs.* brain lysate in healthy donors.** Spearman’s correlation coefficient (*r*) and the corresponding significance (*P*) are displayed in the graph.

**
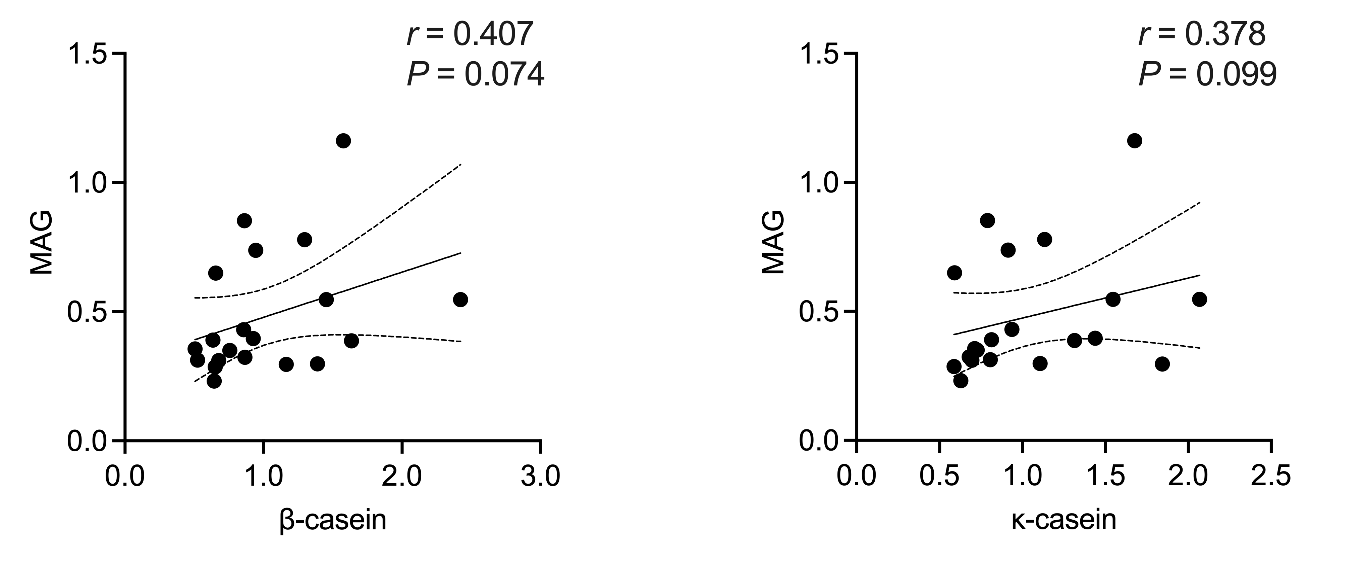
**

**Figure S4 Correlation between β- and κ-casein *vs.* brain lysate in healthy donors.** Spearman’s correlation coefficient (*r*) and the corresponding significance (*P*) are displayed in the graph. MAG, myelin-associated glycoprotein.
